# Ultrasound features help identify patients who can undergo noninvasive management for suspected retained products of conception: a single institutional experience

**DOI:** 10.1007/s00261-020-02948-y

**Published:** 2021-01-18

**Authors:** Shrilakshmi Vyas, Hailey H. Choi, Sara Whetstone, Priyanka Jha, Liina Poder, Dorothy J. Shum

**Affiliations:** 1 Dept of Radiology and Biomedical Imaging, University of California San Francisco, 505 Parnassus Avenue, Floor 02, Room 255, Box 0628, San Francisco, CA 94117 USA; 2 Dept of Obstetrics and Gynecology, University of California San Francisco, 505 Parnassus Avenue, San Francisco, CA 94117 USA

**Keywords:** Retained products of conception, Enhanced myometrial vascularity, Postpartum hemorrhage, Uterine arteriovenous malformation, Pelvic ultrasound

## Abstract

**Objectives:**

To evaluate ultrasound (US) features associated with successful noninvasive management for suspected retained products of conception (RPOC).

**Methods:**

In this IRB-approved retrospective study, the radiology report database was queried for pelvic US with keywords of postpartum hemorrhage (PPH) and/or RPOC over a 2-year period. Follow-up exams, US exams without clinical follow-up, suboptimal image quality, and > 1 year from delivery or pregnancy termination were excluded. Charts were reviewed for clinical presentation and management. Two radiologists reviewed images for endometrial thickness, endometrial echogenicity, endometrial vascularity, and enhanced myometrial vascularity (EMV), as well as inner myometrial peak systolic velocity (PSV) and resistive index (RI) where available. Features were assessed for associations with management approach, and test characteristics were calculated.

**Results:**

Initial query yielded 196 exams, and 48 were excluded. A total of 148 patients were included. Mean age was 34.2 years (21–47), and mean time from delivery or pregnancy termination was 40.4 days (0–223). 81 (55%) underwent noninvasive management: 72 (48%) expectant and 9 (6%) medical. 67 (45%) underwent invasive management: 60 (41%) surgical and 7 (5%) uterine artery embolization. There was substantial inter-reader agreement for assessment of EMV (*K* = 0.78) and endometrial vascularity (*K* = 0.72). Thin endometrial stripe, avascular endometrium, and absence of EMV were associated with successful noninvasive management (*p* < 0.05). Thin endometrium (< 10 mm) had specificity (90%), PPV (88%), and likelihood ratio (5.91) in predicting successful noninvasive management.

**Conclusion:**

Endometrial thickness < 10 mm, avascular endometrium, and absence of EMV are the sonographic features associated with successful noninvasive management for PPH or suspected RPOC.

## Introduction

Postpartum hemorrhage (PPH) is a common cause of severe maternal morbidity and mortality worldwide, with an approximate yearly incidence of 3% in the United States [1]. While there are multiple underlying etiologies for PPH, some of the most common include uterine atony, genital trauma, coagulopathies, placenta accreta spectrum disorder (PASD), and retained products of conception (RPOC) [1]. Primary PPH occurs within 24 h of delivery, while secondary PPH occurs between 24 h and 12 weeks after delivery. RPOC accounts for 30% of secondary PPH [2]. Other causes include subinvolution of the placental implantation site, endometritis, and uterine artery pseudoaneurysm [3]. Imaging often plays a crucial role in the diagnostic workup. Up to 85% of patients with secondary PPH are evaluated with diagnostic pelvic ultrasound (US) at presentation [2]. Postpregnancy bleeding is an all-inclusive term to include bleeding after a term vaginal delivery, Cesarean section, spontaneous abortions, and both medical and surgical terminations.

Diagnostic workup and management of secondary PPH is challenging, as there are often overlapping clinical presentations between normal postpartum bleeding (lochia) versus pathologic bleeding [4]. Beta-HCG levels may not be helpful in confirming the presence of RPOC as it can remain elevated for a period following uncomplicated miscarriage and be subthreshold in cases with necrotic RPOC [5–7] and PASD. Imaging and interpretation of the postpartum uterus is also complicated by its variable appearance and overlap between normal and abnormal states [4, 8–10]. For example, RPOC can present as an endometrial soft tissue mass with or without vascularity [3]. When RPOC is devascularized, its appearance can be indistinguishable from nonpathological avascular postpartum blood products within the endometrial cavity. Furthermore, distinction between simple RPOC and RPOC in the setting of PASD, which may require surgical intervention, can be difficult [8].

In addition, there is inconsistency and confusion in the literature with respect to vascular findings found in the postpregnancy uterus. Terms such as arteriovenous malformations (AVM) and enhanced myometrial vascularity (EMV) have been used interchangeably in the past to describe similar findings which contribute to the challenge of clinical management and can invoke unnecessary procedures such as uterine artery embolization (UAE). EMV refers to a focus of increased vascularity in the myometrium which extends to the endometrium and is seen in the postpregnancy state. It is believed to correspond to subinvolution of the placental implantation site, which can be confirmed histologically by the presence of extravillous trophoblasts and expanded vessels due to delayed conversion back to pre-gravid small-caliber maternal spiral arteries [3, 11]. It may resolve spontaneously or after removal of the RPOC [11–13].

Treatment strategies for PPH range from conservative to more invasive procedural or surgical maneuvers [11, 12, 14, 15]. Conservative management includes both expectant and medical treatment with uterotonic medications, such as misoprostol, or methotrexate. Invasive options include dilation and curettage (D&C), UAE, and hysterectomy. As of now, there is no standardized protocol for the treatment approach of PPH. The decision to treat patients with a surgical approach such as D&C is a careful balance of risk and benefits, often taking into account patient preference and potential complications from either expectant management or surgical intervention. In severe cases with potentially life-threatening hemorrhage, D&C, UAE, or surgical hysterectomy could be warranted [11, 12, 14, 15]. Invasive management approaches run the risk of procedure-related complications such as Asherman syndrome, vascular injuries, radiation exposure, potential negative fertility implications, side effects from anesthesia, and increased cost.

The aim of this study is to evaluate clinical and sonographic findings associated with management approach, in order to identify those that can safely predict successful noninvasive management of PPH and thus help reduce procedure-related complications.

## Methods

### Inclusion and exclusion criteria

**Fig. 1:**
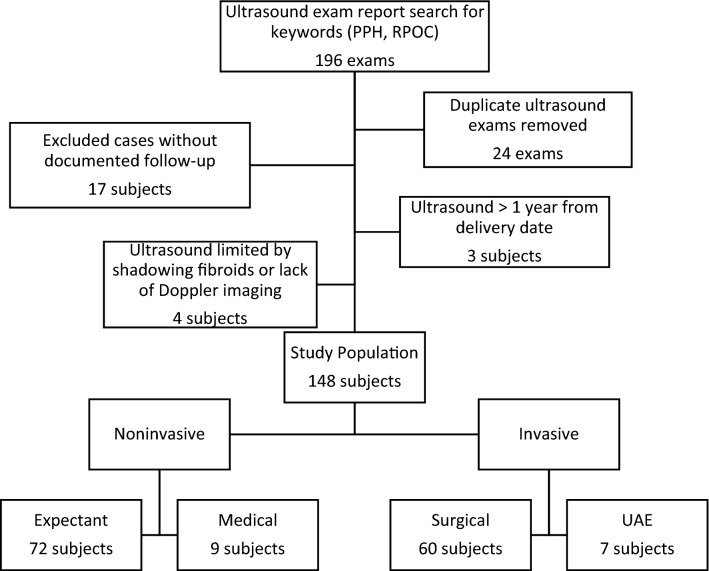
Flowchart for study inclusion and exclusion criteria and categorization of management approaches. *PPH* postpartum hemorrhage, *RPOC* retained products of conception

In this IRB-approved, retrospective study, the radiology report database was queried for pelvic US reports containing keywords of “postpartum hemorrhage” and/or “retained products of conception” over a 2-year interval (6/1/2017–6/30/2019). For patients with multiple exams, US exam at initial postpregnancy presentation was assessed (Fig. 1). Patients without clinically documented follow-up were excluded. US exams that occurred more than 1 year from delivery date or pregnancy termination and those with significant technical limitations were also excluded.

### Clinical chart review

Selective chart review was conducted for clinical parameters at presentation including time from delivery or pregnancy termination, gestational age at time of delivery, delivery method, clinical assessment of hemodynamic stability, hemoglobin levels, blood pressure, heart rate, and beta-HCG levels. Resulting clinical management—expectant, medical, surgical (D&C or hysterectomy), and UAE by interventional radiology (IR)—was recorded as clinical outcomes. Patients who underwent successful noninvasive management had documented resolution of symptoms. For a subset of cases that underwent more than one management approach, the most invasive method was designated as the final outcome. Impression from the original US report was categorized as (a) no evidence of RPOC or (b) possible or definite RPOC.

### US technique

US examinations were performed by American Registry for Diagnostic Medical Sonography (ARDMS) certified sonographers on GE Logiq E9 (GE Healthcare, Waukesha, WI) or Siemens S2000 (Siemens Medical Solutions, Mountain View, CA) units. Transabdominal images were obtained using convex or linear probes spanning 1–6 MHz and 2–8 MHz frequencies, respectively, with grayscale and color Doppler technique. Patients then underwent transvaginal imaging of the uterus using probes spanning 3–10 MHz frequencies with grayscale and color Doppler technique, unless declined by the patient. Power and spectral Doppler evaluation were not routinely performed per institution protocol but were obtained as deemed necessary by the sonographer and/or radiologist at the time of the examination.

### Imaging review

**Fig. 2:**
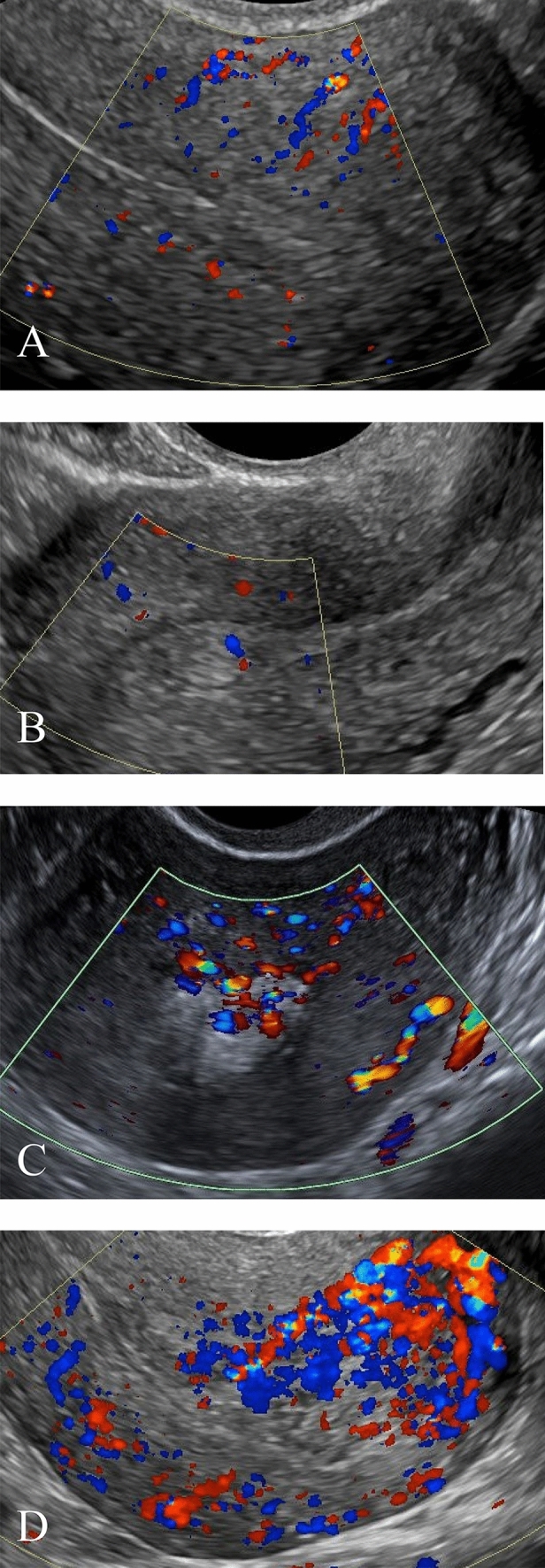
**a** 34-year-old female with vaginal bleeding following spontaneous abortion at gestational age of 5 weeks and 6 days with avascular endometrium (score 0) on pelvic US. Patient underwent expectant management with successful resolution of symptoms. **b** 34-year-old female with pelvic pain and vaginal bleeding following D&C at 5 weeks and 2 days gestational age with mild endometrial vascularity (score 1) on pelvic US. Patient underwent expectant management with successful resolution of symptoms. **c** 39-year-old female with vaginal bleeding following vaginal delivery at gestational age of 37 weeks and 1 day with moderate endometrial vascularity (score 2). Patient underwent surgical management with D&C. Pathology confirmed the presence of RPOC, including degenerating RPOC. **d** 35-year-old female with fever, abdominal pain, and nausea following D&C at 11 weeks and 0 days gestational age with marked endometrial vascularity (score 3). Patient underwent surgical management via repeat D&C. Pathology confirmed the presence of RPOC

**Fig. 3:**
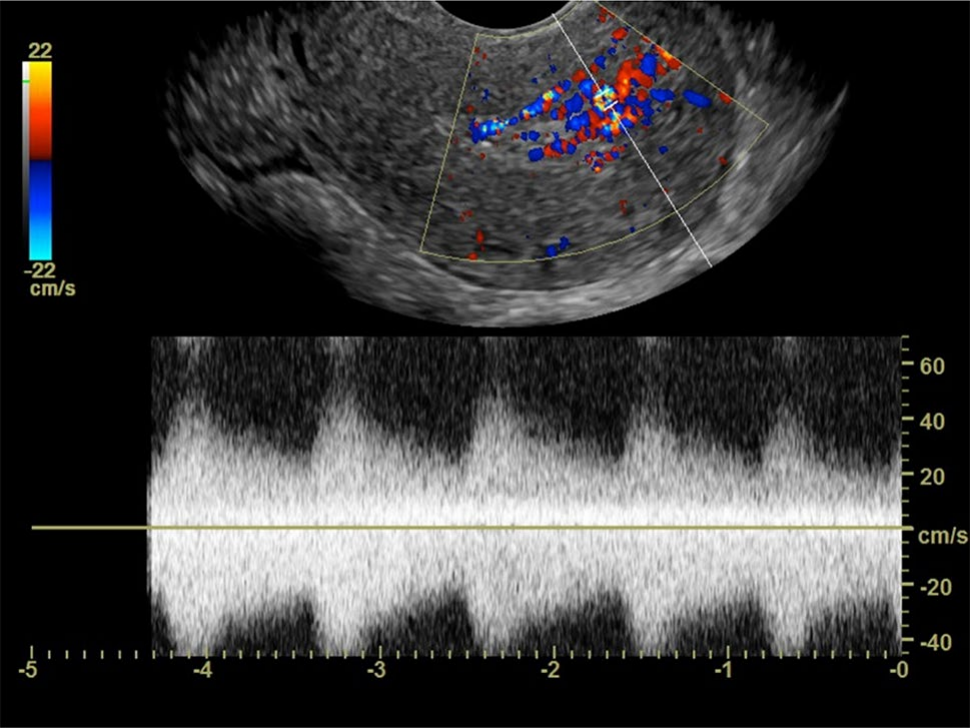
25-year-old female who presented with abnormal bleeding 6 weeks after D&C at 9 weeks 2 days of gestational age. US image of the uterus in longitudinal plane with color and spectral Doppler interrogation shows arterial waveforms within the inner myometrium, with a peak systolic velocity of 56 cm/s and resistive index of 0.46 (measurements not shown). Patient underwent an uncomplicated D&C for RPOC. Pathology confirmed degenerating RPOC and placental implantation site

**Fig. 4:**
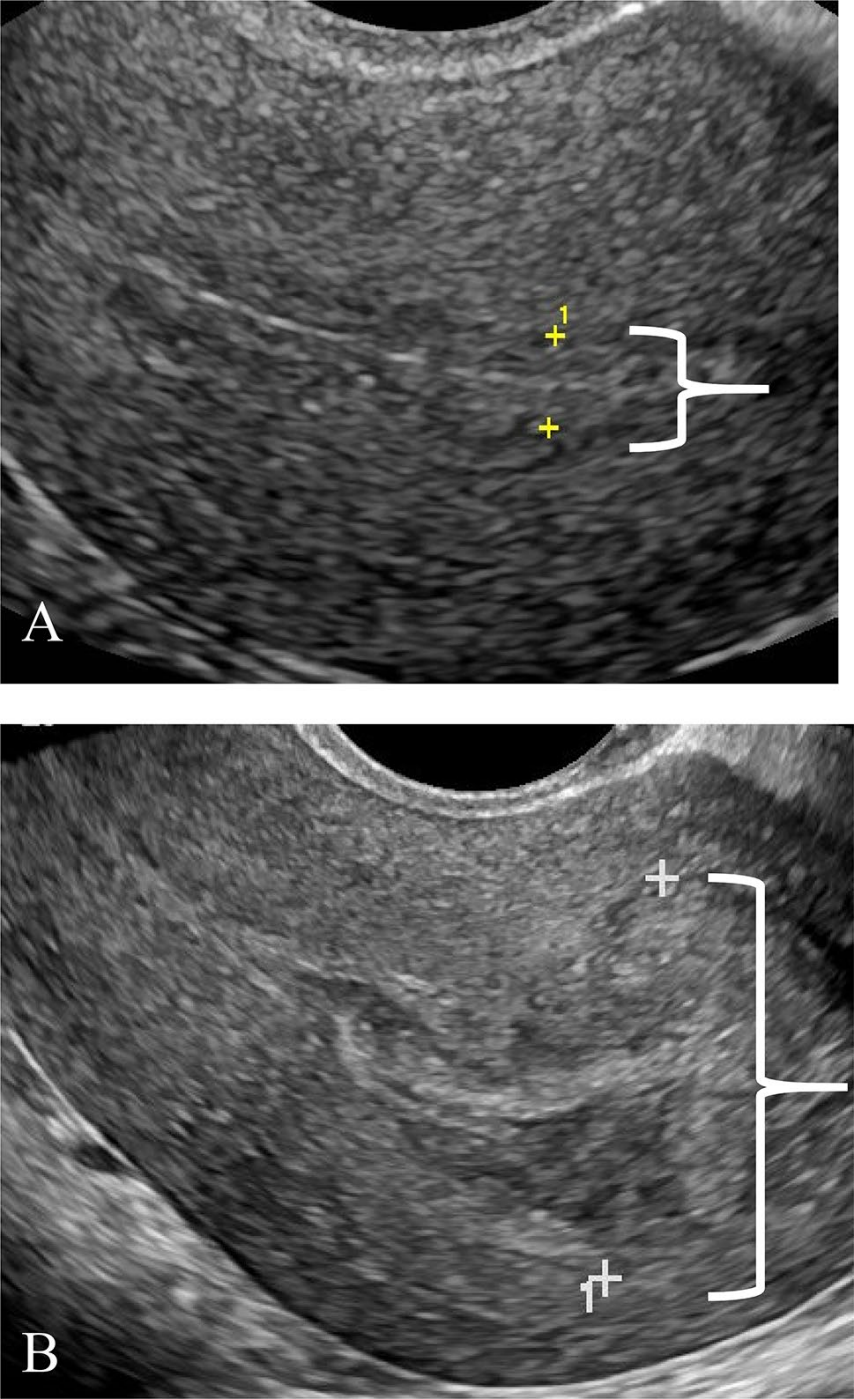
**a** 34-year-old female who presented with vaginal bleeding after spontaneous abortion at 5 weeks and 6 days gestational age. Pelvic US of the endometrium in the longitudinal plane demonstrates a thin endometrial stripe (calipers). Patient underwent expectant management with successful resolution of symptoms. **b** 35-year-old female (same patient as in Fig. 2d) with fever, abdominal pain, and nausea who had undergone D&C at gestational age of 11 weeks and 0 days. Pelvic US of the endometrial stripe in the longitudinal plane shows a thick endometrium (calipers). Patient underwent a repeat D&C. Pathology confirmed the presence of RPOC

**Fig. 5:**
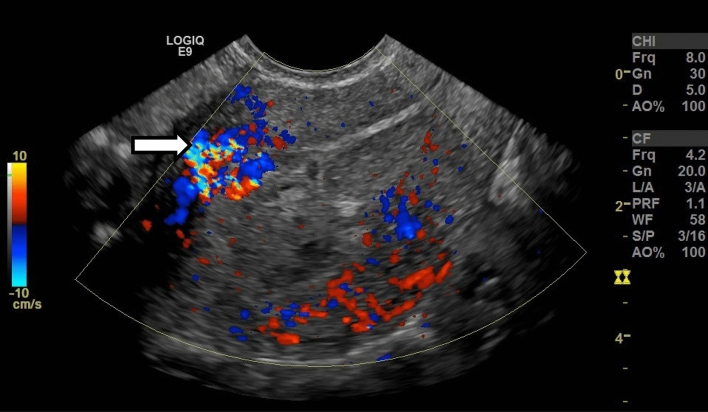
Enhanced myometrial vascularity in a 28-year-old female who presented with vaginal bleeding following D&C at 5 weeks of gestational age. Color Doppler US image of the uterus in longitudinal plane demonstrates focally increased color Doppler signal spanning the anterior myometrium (arrow). The color signal extends to the endometrium, which is distended with isoechoic material. Patient was treated medically with uterotonic agents, and symptoms were resolved subsequently

US images were retrospectively and independently reviewed by 2 attending radiologists who were fellowship-trained in body imaging with 2 and 8 years of post-fellowship experience. In cases of disagreement, a third reader with 15 years of post-fellowship experience also reviewed the cases. US features analyzed include endometrial thickness, endometrial echogenicity, and vascularity (Types 0–3, denoting avascular, mild, moderate, or marked, as established by a prior study, Fig. 2) [16], inner myometrial peak systolic velocity (PSV) and resistive index (RI) (Fig. 3), and the presence of EMV. Endometrial thickness was measured in standard fashion, anteroposterior dimension in the longitudinal plane (Fig. 4). EMV was defined as a focal area of increased vascularity spanning the full myometrial thickness and extending to the endometrium, based on description by previous studies [17] (Fig. 5).

### Statistical analysis

For data analysis, clinical management strategies were grouped into noninvasive (expectant or medical management) versus invasive (surgical and UAE). Statistical analysis was performed with R. Interobserver variability was assessed using the Landis and Koch scale: kappa values of 0–0.20 were considered poor agreement; 0.21–0.40, fair; 0.41–0.60, moderate; 0.61–0.80, substantial; and 0.81–1.0, almost perfect agreement [18]. Weighted kappa was used to assess for agreement in endometrial vascularity score, and Cohen’s kappa was used to assess for the presence or absence of EMV. Fisher’s exact and Mann–Whitney *U* tests were used to assess for correlation with management strategies for categorical and numerical variables, respectively. For factors with significant association, sensitivity, specificity, positive predictive value (PPV), negative predictive value (NPV), and likelihood ratio were calculated. Receiver operating characteristic analysis was performed for endometrial thickness.

## Results

### Study population

Initial query yielded 196 cases. 24 cases were duplicate exams and were excluded. 17 subjects did not have follow-up documentation and were excluded. 3 patients presented greater than 1 year after delivery and were excluded. 4 exams were limited by acoustic shadowing from fibroids or did not have Doppler evaluation and were excluded. Final population included 148 subjects. Mean age was 34.4 years (range 21–47), mean time from end of pregnancy was 40.4 days with a range of 0–223 days. Delivery and/or pregnancy termination method varied, with the most common method being vaginal delivery (48 cases, or 32.4%) (Table 1).

**Table 1:** Clinical parameters for each management group

	Noninvasive	Invasive	Overall	*p*-value
Age	33.8 (21–47)	35.2 (21–47)	34.2 (21–47)	0.099
Time from delivery (days)	39.1 (0–215)	42.1 (1–223)	40.4 (0–223)	0.783
Gestational age at time of delivery (weeks)	22.3 (1.4–42.0)	25.9 (6–41.9)	23.9 (1.4–42.0)	0.093
Hemoglobin (g/dl)^a^	12.5 (8.6–15.5)	11.5 (7.1–13.7)	12.0 (7.1–15.5)	**0.009**
Heart rate (beats per minute)^b^	76.3 (56–115)	77.4 (52–110)	76.8 (52–115)	0.563
Systolic blood pressure (mmHg)^c^	114 (91–138)	113 (90–163)	114 (90–163)	0.443
Diastolic blood pressure (mmHg)^c^	68.7 (52–90)	67.5 (46–96)	68.1 (46–96)	0.680
Beta-HCG (mIU/mL)^d^	903 (0–19,300)	438 (0–6680)	667 (19,300)	0.401
Delivery method				**0.013**
Cesarean section	16 (19.8%)	9 (13.4%)	26 (16.9%)	
D&C	28 (34.6%)	15 (23.4%)	43 (29.1%)	
Medical abortion	5 (6.2%)	8 (11.9%)	13 (8.8%)	
Spontaneous abortion	1 (17.3%)	5 (7.5%)	19 (12.8%)	
Vaginal delivery	18 (22.2%)	30 (44.8%)	48 (32.4%)	

Mean values and range are presented. For delivery method, number of cases and percentages are presented

*D&C* dilatation and curettage

^a^Hemoglobin levels were available in 87 cases (42 in noninvasive group, 45 in invasive group)

^b^Heart rate at presentation was available in 114 cases

^c^Blood pressure at presentation was available in 128 cases

^d^beta-HCG levels were available in 57 cases (28 in noninvasive group, 29 in invasive group)

Bold value indicates statistical significant *p* < 0.05

### Clinical outcomes (management)

Of the 148 patients, 81 (55%) underwent noninvasive management: 72 (48%) expectant management and 9 (6%) medical treatment with uterotonic agents and/or methotrexate. 67 (45%) underwent invasive management: 60 (41%) surgical treatment and 7 (5%) UAE (Fig. 1). For surgical treatment, almost all cases underwent D&C, except for 1 patient who underwent hysterectomy for suspected placenta accreta. 7 patients underwent UAE—3 for questioned AVM, 2 for excessive bleeding after D&C, 1 for suspected PASD, and 1 for suspected pseudoaneurysm; no AVM was found at angiography. 56 out of 66 (86.2%) patients with original US interpretation of no RPOC underwent noninvasive management. 56 of 81 (69.1%) patients with initial US impression of RPOC underwent invasive management. In 1 patient, the initial US was interpreted as nondiagnostic for the lack of transvaginal imaging.

### Clinical parameters

Clinical parameters for invasive versus noninvasive outcomes are summarized in Table 1. Of the clinical parameters, the distribution of delivery methods was statistically different (*p* < 0.05), with D&C being most prevalent in the noninvasive group, versus vaginal delivery in the invasive group. Hemoglobin levels were slightly higher in the noninvasive group (*p* < 0.05), but with significant overlap. Time from delivery, gestational age at delivery, heart rate, systolic and diastolic blood pressure, and beta-HCG were not significantly different. Beta-HCG was only available in 57 patients, as it is not routinely obtained in the evaluation of RPOC by our referring providers. On chart review, only 2 patients were noted to have clinical signs of hemodynamic instability: 1 patient subsequently underwent UAE with gelfoam embolization, and 1 patient’s condition stabilized, allowing for expectant management.

### Imaging features

Interobserver variability was assessed for endometrial vascularity score and the presence or absence of EMV. Substantial agreement was seen for the assessment of EMV (*K* = 0.78). Substantial agreement was seen for endometrial vascularity score (*K* = 0.72).

**Fig. 6:**
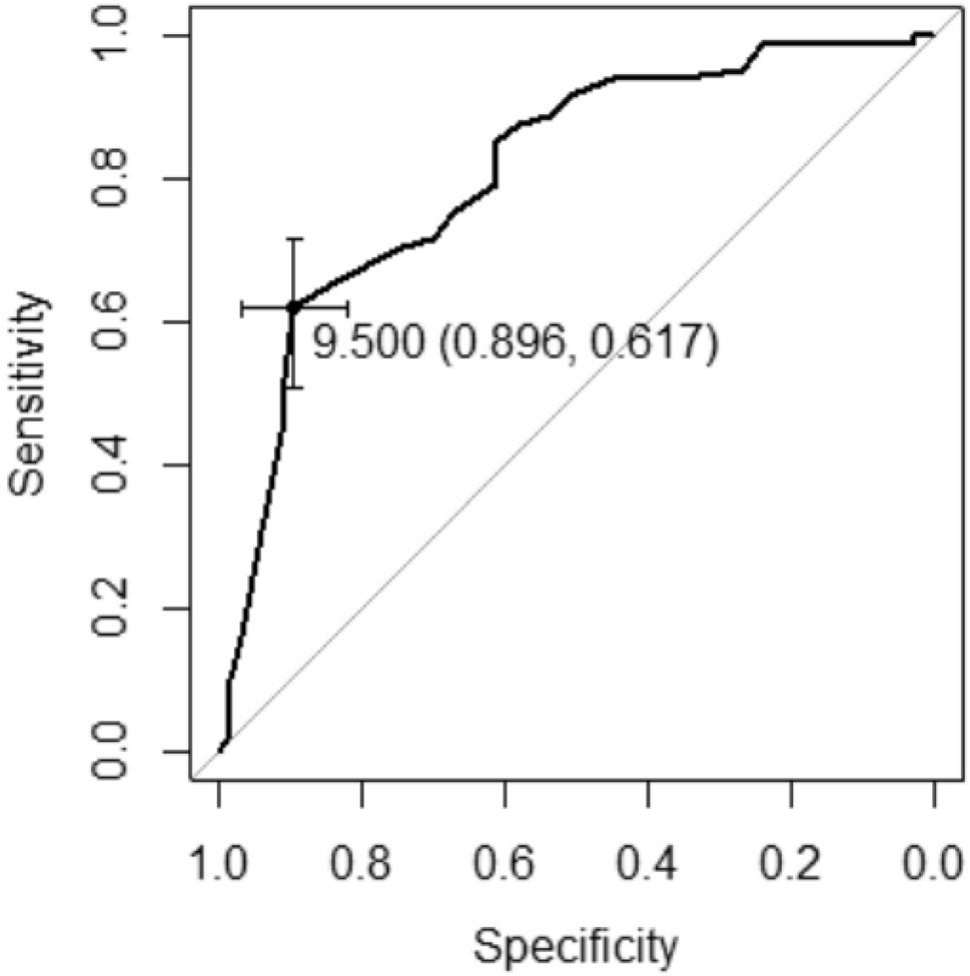
Receiver operating characteristic curve for endometrial thickness as a predictor for noninvasive management. Area under the curve (AUC) is 0.809. Best threshold by Youden method was 9.5 mm, with sensitivity of 61.7% and specificity of 89.6%

Imaging features per treatment group are summarized in Table 2. The endometrial stripe was significantly thinner for the noninvasive management group with a mean of 9.23 mm, versus 19.2 mm *p* (*p* < 0.05). Receiver operating characteristic analysis revealed an area under the curve of 0.809; best threshold by Youden method of 9.5 mm, with sensitivity of 61.7% and specificity of 89.6% in predicting noninvasive management (Fig. 6). The absence of endometrial vascularity and EMV were observed more often in the group managed conservatively (*p* < 0.05). No significant difference was observed for endometrial echogenicity. Spectral Doppler was only acquired for 43 of patients to allow for PSV and RI measurement. There was significant overlap in the PSV and RI between the invasively managed versus noninvasively managed groups.

**Table 2:** Summary of imaging features for each management group

	Noninvasive	Invasive	Overall	*p*-value
Endometrial thickness (mm)^a^	9.23 (1–41) IQR: 4–13	19.2 (1–78) IQR: 12–23	13.8 (1–78)	** < 0.001**
< 10 mm	50 (61.7%)	7 (10.4%)	57 (38.5%)	** < 0.001**
≥ 10 mm	31 (38.3%)	60 (89.6%)	91 (61.5%)	
Endometrial echogenicity				0.360
Hyperechoic	37 (45.7%)	37 (55.2%)	74 (50.0%)	
Isoechoic	43 (53.1%)	30 (44.8%)	73 (49.3%)	
Hypoechoic	1 (1.2%)	0 (0%)	1 (0.7%)	
Endometrial vascularity				** < 0.001**
0	71 (87.7%)	29 (43.3%)	100 (67.6%)	
1	6 (7.4%)	12 (17.9%)	18 (12.2%)	
2	2 (2.5%)	13 (19.4%)	15 (10.1%)	
3	2 (2.5%)	13 (19.4%)	15 (10.1%)	
Peak systolic velocity (cm/s)^a^	50.6 (14–100)	60.6 (3–252)	58.5 (3–252)	0.919
Resistive index^a^	0.489 (0–0.73)	0.493 (0.17–0.89)	0.492 (0–0.89)	0.411
Enhanced myometrial vascularity^a^				** < 0.001**
Absent	71 (87.7%)	28 (41.8%)	99 (66.9%)	
Present	10 (12.3%)	39 (58.2%)	49 (32.1%)	

Mean values and ranges are presented for endometrial thickness, peak systolic velocity, and resistive index. Number of cases and percentages are presented for endometrial echogenicity, endometrial vascularity, and enhanced myometrial vascularity

*IQR* interquartile range

^a^Peak systolic velocity and resistive index could be measured in 43 cases (9 in noninvasive group, 34 in invasive group)

Bold value indicates statistical significant *p* < 0.05

Endometrial thickness, endometrial vascularity type, and the presence of EMV were the imaging features significantly associated with management approach. Sensitivity, specificity, PPV, NPV, and likelihood ratios were calculated for each of these features (Table 3). Endometrial stripe was considered thin if it was measured less than 10 mm. Endometrial vascularity was considered absent for type 0, and present for types 1–3. Of the three features, endometrial thickness demonstrated the highest specificity of 90%, PPV of 88%, and positive likelihood ratio of 5.91 in predicting successful noninvasive management. In combination, the three features of thin endometrium, absence of endometrial vascularity, and absence of EMV yielded a PPV of 90%, specificity 93%, and likelihood ratio 7.78.

**Table 3:** Sensitivity, specificity, positive predictive value, and negative predictive value of three imaging features in predicting successful noninvasive management (expectant or medical treatment)

	Thin endometrium < 10 mm	Absence of endometrial vascularity (0 vs. 1–3)	Absence of EMV	All three features favoring noninvasive management	At least two features favoring noninvasive management
Sensitivity	62%	88%	88%	58%	85%
Specificity	90%	57%	58%	93%	61%
Positive predictive value	88%	71%	72%	90%	73%
Negative predictive value	66%	79%	80%	65%	77%
Positive likelihood ratio (95% confidence interval)	5.91 (2.87–12.16)	2.03 (1.52–2.70)	2.10 (1.56–2.81)	7.78 (3.28–18.43)	2.20 (1.60–3.01)

*EMV* enhanced myometrial vascularity

### Subset of cases with pathologic confirmation

Pathology results were available in 58 cases; in two patients, tissue from D&C was not sent for formal pathologic evaluation. 12 were negative for RPOC; 46 were positive. Of the imaging features, greater endometrial thickness and EMV were significantly associated with pathology-proven RPOC (*p* < 0.05) (Table 4). Using 10 mm as the cut-off for endometrial thickness, thick endometrial stripe was 98% sensitive for RPOC on pathology, with PPV of 87% and NPV of 83%; however, it demonstrated poor specificity of 42%. In three cases, pathology revealed RPOC with placenta accreta.

**Table 4:** Correlation of imaging features and the presence or absence of RPOC on pathology

	RPOC	No RPOC	Overall	*p*-value
Endometrial thickness (mm)	21.3 (9.0–78.0)	12.8 (1.0–50.0)	19.3 (1.0–78.0)	**0.002**
Endometrial echogenicity				**0.048**
Hyperechoic	22 (47.8%)	10 (83.3%)	32 (55.2%)	
Isoechoic	24 (52.2%)	2 (16.7%)	26 (44.8%)	
Endometrial vascularity type				**0.162**
0	16 (34.8%)	9 (75.0%)	285(43.1%)	
1	11 (23.9%)	1 (8.3%)	12 (20.7%)	
2	10 (21.7%)	1 (8.3%)	11 (19.0%)	
3	9 (19.6%)	1 (8.3%)	10 (17.2%)	
Enhanced myometrial vascularity^a^				**0.018**
Absent	15 (32.6%)	9 (75.0%)	24 (41.4%)	
Present	31 (67.4%)	3 (25.0%)	34 (58.6%)	

Means and ranges are presented for endometrial thickness. Number of cases and percentages are presented for endometrial echogenicity, endometrial vascularity type, and enhanced myometrial vascularity

*RPOC* retained products of conception

Bold value indicates statistical significant *p* < 0.05

## Discussion

In our single institution experience of patients presenting with suspected RPOC, imaging findings were better predictors of conservative management than clinical features. Endometrial thickness < 10 mm, absence of endometrial vascularity, and absence of EMV were the sonographic findings that predicted successful management without procedural intervention and thus presumably represented patients who either did not have RPOC or had mild disease that could be managed conservatively. When combined, these 3 findings predicted successful conservative management with 93% specificity and 90% PPV.

Of the 3 imaging features that most significantly correlated with management strategies, thin endometrial stripe proved the most useful. Durfee et al. previously concluded that a thin endometrial stripe, less than 10 mm, without an endometrial mass or fluid excludes the presence of RPOC [19]. This cut-off of 10 mm was closely aligned with our threshold value of 9.5 mm on ROC analysis (AUC 0.809, specificity of 89.6%) and resulted in a high specificity of 90% and PPV 88%. This supports that noninvasive management can be pursued for endometrial thicknesses less than 10 mm. It should be noted that the endometrial thickness cut-off should only be applied to symptomatic patients in the appropriate clinical setting, as the endometrial stripe can measure up to 16 mm in normal premenopausal women [20].

Endometrial vascularity was assessed by implementing a grading system as defined by Kamaya et al. [16]. By stratifying endometrial vascularity to avascular (type 0) or vascular (types 1–3), absence of endometrial vascularity yielded a specificity 57%, and PPV 71%, lower than those of endometrial thickness. Our study results are similar with Kamaya et al. whereby increased endometrial stripe thickness and endometrial vascularity scores were predictive of invasive management, with an odds ratio of 1.08 and 1.77, respectively [21]; in their experience, the presence of any vascularity in the endometrium (types 1–3) had a high likelihood of representing RPOC, with a PPV of 96%. This is in comparison to our experience where just under half (43.3%) of the patients who underwent invasive management had no detectable vascularity within the endometrial stripe; in the subset of cases with available pathology results, 34.8% of pathology-proven RPOC cases did not demonstrate endometrial vascularity (type 0 avascular). While endometrial vascularity may be a helpful predictive measure, the absence of vascularity did not exclude RPOC and did not deter D&C or other procedures for management.

Absent EMV performed similarly to absent endometrial vascularity in predicting successful noninvasive management with a sensitivity of 88%, low specificity 58%, and moderate PPV of 72%. The presence of EMV was correlated with more invasive management approaches. Previous work has shown EMV to be a common transient finding in postpregnancy patients [17]. In fact, three cases from our study with EMV did not have pathologic evidence of RPOC, which supports that EMV reflects isolated placental site subinvolution or EMV persistence from minute quantities of RPOC which can resolve without intervention. More commonly, EMV was associated with pathology-proven RPOC (67.4%) and is thought to reflect preserved vascular supply to the retained tissue [22]. RPOC likely inhibits the involution of the placental site.

In our experience, most of the positive EMV cases were successfully managed conservatively or with surgical removal. No AVM or fistula was found in the 7 cases that underwent diagnostic angiography. While other authors [12] have previously suggested an EMV velocity cut-off to guide surgical versus embolization management, we found significant overlap in both PSV and RI measurements between patients who were managed expectantly versus those who required surgical or angiographic measures. Our findings are concordant with other published data (with the largest sample size to date of 43 patients, to our knowledge) showing that there is no correlation between PSV and clinical management [11, 17]. While the high flow, low resistance waveform pattern of EMV is difficult to distinguish from other types of vascular malformations based on imaging, it is our proposal that in the postpregnancy state, these commonly encountered myometrial vascular changes seen in isolation or associated with RPOC should be referred to as EMV to avoid mismanagement and confusion with the exceedingly rare true congenital AVM or post-procedure AVF/AVM spectrum.

Further studies are needed to assess longitudinal patient outcomes and the natural progression of imaging and clinical findings in such cases. In our experience, there was substantial inter-reader agreement for the assessment of EMV and endometrial vascularity grading, which was not previously reported [11, 17, 21, 23]. Few cases with inter-reader variability may have been affected by technical parameters at the time of image acquisition such as color scale and color gain. Adopting standardized imaging technique and interpretation can facilitate future research.

Limitations of our study include its retrospective design and small sample size. Some parameters were not consistently available for analysis due to the retrospective design. For example, PSV and RI are not routinely performed in our standard protocol for pelvic sonography. Only a small subset of patients had beta-HCG information as our institution’s referring providers do not use this data to guide management for suspected RPOC. In fact, we had 4 patients with subthreshold beta-HCG levels (< 5 mIU/mL) but had RPOC by pathology. If there is imaging or clinical suspicion for gestational trophoblastic disease (none in our study population), beta-HCG evaluation is helpful. Pathological analysis from tissue sampling was only available for a subset of patients. We also recognize there is a significant selection bias, as only patients with significant concerns of PPH or suspicion for RPOC were referred to radiology, and only reports containing keywords of “PPH” or “R/O RPOC” were analyzed. This would incur a selection bias for patients with mild and moderate symptoms, stable enough to obtain an ultrasound, and exclude those with severe symptoms who are treated immediately without formal imaging by the Radiology Department. This, however, closely simulates clinical radiology practice, as radiologists often only receive referrals for cases where management may change based on imaging findings. Our keyword-based search resulted in a heterogeneous study population, including cases with suspected endometritis or PASD. Our study is also significantly impacted by variability in clinical management; for example, personal preference from both provider and patient and/or socioeconomic factors may have driven management decisions. The original US interpretation also likely influenced treatment approach.

RPOC remains a challenging clinical and imaging diagnosis to make as shown by our pathology sub-analysis. Our results indicate that not all patients who underwent D&C had pathology-proven RPOC. Thus defining those US features which lead to successful noninvasive management helps avoid unnecessary procedure-related complications. Determination of what imaging findings truly necessitate an intervention was not possible based on the retrospective nature of our study. However, through chart review, we were able to identify those imaging findings most associated with conservative management without need for subsequent intervention.

## Conclusion

Thin endometrial stripe < 10 mm is a useful criterion to predict successful noninvasive management in patients with suspected RPOC. The absence of endometrial vascularity and EMV were also associated with conservative management, but to a slightly lesser degree. While a vascular endometrium was more frequently intervened on, its absence did not exclude RPOC. RPOC is a difficult diagnosis to make based on clinical and imaging findings. In such challenging situations, US can play a key role by identifying patients who can undergo expectant or medical treatment.

## References

[1] Marshall AL, Durani U, Bartley A, et al. The impact of postpartum hemorrhage on hospital length of stay and inpatient mortality: a National Inpatient Sample–based analysis. *Am J Obstet Gynecol*. 2017;217(3):344.e1–344.e6. 10.1016/j.ajog.2017.05.004 10.1016/j.ajog.2017.05.004 28502758

[2] Dossou M, Debost‐Legrand A, Déchelotte P, Lémery D, Vendittelli F. Severe Secondary Postpartum Hemorrhage: A Historical Cohort. *Birth*. 2015;42(2):149–155. 10.1111/birt.12164 10.1111/birt.12164 25867033

[3] Wang SS, Shum D, Kennedy A. Imaging of Postpartum/Peripartum Complications. *Radiol Clin North Am*. 2020;58(2):431–443. 10.1016/j.rcl.2019.10.007 10.1016/j.rcl.2019.10.007 32044016

[4] Achiron R, Goldenberg M, Lipitz S, Mashiach S. Transvaginal Duplex Doppler Ultrasonography in Bleeding Patients Suspected of Having Residual Trophoblastic Tissue. *Obstet Gynecol*. 1993;81(4):507–511. 8459957

[5] Fernandez, C., Braginsky, L., Levine, E., Locher, S. and Sodini, I. (2014), OP24.10: Retained products of conception coincident with negative urine hCG: a case series. Ultrasound Obstet Gynecol, 44: 141–141. 10.1002/uog.13875

[6] Antonella G, Luisa DB, Chiara A, Alessandra R, Caserta D. Conservative and timely treatment in retained products of conception: a case report of placenta accreta ritention. :5. PMC4680531 26722586

[7] Carusi, Daniela. Retained products of conception. In: UpToDate, Post, TW (Ed), UpToDate, Waltham, MA, 2020.

[8] Mulic‐Lutvica A, Bekuretsion M, Bakos O, Axelsson O. Ultrasonic evaluation of the uterus and uterine cavity after normal, vaginal delivery. *Ultrasound Obstet Gynecol*. 2001;18(5):491–498. 10.1046/j.0960-7692.2001.00561.x 10.1046/j.0960-7692.2001.00561.x 11844171

[9] Sadan O, Golan A, Girtler O, et al. Role of Sonography in the Diagnosis of Retained Products of Conception. *J Ultrasound Med*. 2004;23(3):371–374. 10.7863/jum.2004.23.3.371 10.7863/jum.2004.23.3.371 15055784

[10] Plunk M, Lee JH, Kani K, Dighe M. Imaging of Postpartum Complications: A Multimodality Review. *Am J Roentgenol*. 2013;200(2):W143-W154. 10.2214/AJR.12.9637 10.2214/AJR.12.9637 23345378

[11] Timor-Tritsch IE, Haynes MC, Monteagudo A, Khatib N, Kovács S. Ultrasound diagnosis and management of acquired uterine enhanced myometrial vascularity/arteriovenous malformations. *Am J Obstet Gynecol*. 2016;214(6):731.e1–731.e10. 10.1016/j.ajog.2015.12.024 10.1016/j.ajog.2015.12.024 26873276

[12] Timmerman D, Wauters J, Calenbergh SV, et al. Color Doppler imaging is a valuable tool for the diagnosis and management of uterine vascular malformations. *Ultrasound Obstet Gynecol*. 2003;21(6):570–577. 10.1002/uog.159 10.1002/uog.159 12808674

[13] Jain, K. and Fogata, M. (2007), Retained products of conception mimicking a large endometrial AVM: Complete resolution following spontaneous abortion. J. Clin. Ultrasound, 35: 42–47. 10.1002/jcu.20250 10.1002/jcu.20250 17024675

[14] Groszmann YS, Murphy ALH, Benacerraf BR. Diagnosis and management of patients with enhanced myometrial vascularity associated with retained products of conception. *Ultrasound Obstet Gynecol*. 2018;52(3):396–399. 10.1002/uog.18954 10.1002/uog.18954 29124818

[15] Hoveyda F, MacKenzie IZ. Secondary postpartum haemorrhage: incidence, morbidity and current management. *BJOG Int J Obstet Gynaecol*. 2001;108(9):927–930. 10.1111/j.1471-0528.2001.00230.x 10.1111/j.1471-0528.2001.00230.x 11563461

[16] Kamaya A, Petrovitch I, Chen B, Frederick CE, Jeffrey RB. Retained Products of Conception. *J Ultrasound Med*. 2009;28(8):1031–1041. 10.7863/jum.2009.28.8.1031 10.7863/jum.2009.28.8.1031 19643786

[17] Bosch TVD, Schoubroeck DV, Lu C, Brabanter JD, Huffel SV, Timmerman D. Color Doppler and gray-scale ultrasound evaluation of the postpartum uterus. *Ultrasound Obstet Gynecol*. 2002;20(6):586–591. 10.1046/j.1469-0705.2002.00851.x 10.1046/j.1469-0705.2002.00851.x 12493048

[18] Landis JR, Koch GG. The measurement of observer agreement for categorical data. *Biometrics*. 1977;33(1):159–174. 843571

[19] Durfee SM, Frates MC, Luong A, Benson CB. The Sonographic and Color Doppler Features of Retained Products of Conception. *J Ultrasound Med*. 2005;24(9):1181–1186. 10.7863/jum.2005.24.9.1181 10.7863/jum.2005.24.9.1181 16123177

[20] Tsuda H, Ito YM, Todo Y, et al. Measurement of endometrial thickness in premenopausal women in office gynecology. *Reprod Med Biol*. 2017;17(1):29–35. 10.1002/rmb2.12062 10.1002/rmb2.12062 PMC5768977 29371818

[21] Kamaya A, Krishnarao PM, Nayak N, Jeffrey RB, Maturen KE. Clinical and imaging predictors of management in retained products of conception. *Abdom Radiol*. 2016;41(12):2429–2434. 10.1007/s00261-016-0954-x 10.1007/s00261-016-0954-x 27853850

[22] Wachter DL, Thiel F, Agaimy A. Subinvolution of the Placental Site 6 Years After Last Delivery. *Int J Gynecol Pathol*. 2011;30(6):581–582. 10.1097/PGP.0b013e318224df3a 10.1097/PGP.0b013e318224df3a 21979595

[23] Grewal K, Al‐Memar M, Fourie H, Stalder C, Timmerman D, Bourne T. The natural history of pregnancy-related enhanced myometrial vascularity following miscarriage. *Ultrasound Obstet Gynecol*. n/a(n/a). 10.1002/uog.21872 10.1002/uog.21872 31503383

